# Intracranial Carotid Artery Aneurysm Treatment: First Reported Case of DERIVO^®^Flow-Diverter Placement by Direct Carotid Artery Puncture

**DOI:** 10.3390/brainsci10050320

**Published:** 2020-05-25

**Authors:** Giuseppe Guzzardi, Bruno Del Sette, Carmelo Stanca, Andrea Paladini, Andrea Galbiati, Marco Spinetta, Massimiliano Cernigliaro, Massimiliano Leigheb, Alessandro Carriero

**Affiliations:** 1 SCDU Department of Interventional and Neurointerventional Radiology, “Maggiore della Carità” University Hospital, 28100 Novara, Italy; brunodelsette@gmail.com (B.D.S.); carmelostanca@gmail.com (C.S.); andreapaladini1988@gmail.com (A.P.); andrea.galbiati.1990@gmail.com (A.G.); marcospinetta90@gmail.com (M.S.); massimiliano.cernigliaro@gmail.com (M.C.); alessandro.carriero@med.uniupo.it (A.C.); 2 Department of Health Sciences, SCDU Orthopedics and Traumatology Institute, “Maggiore della Carità” University Hospital, 28100 Novara, Italy; massimiliano.leigheb@uniupo.it

**Keywords:** brain, neuroradiology, neuroimaging, intracranial aneurysm, internal carotid artery, embolization, flow-diverter, digital subtracted angiography

## Abstract

Brain health may be threatened by aneurysm ruptures, and early recognition of these vascular malformations allows for neuroradiological intervention. Neurointerventional procedures are usually performed with femoral artery access. In patients with severe anatomical complexity of the supra-aortic vessels, however, treatment by this approach could be hindered or impossible. Flow-diverter stent deployment is an effective and safe treatment for large, wide necked intracranial aneurysms, but it requires a complete and firm stability of the coaxial system to achieve a correct and precise deployment of the device. We present the first reported Italian case of a patient with an intracranial aneurysm which was treated with Flow-diverter stent (DERIVO^®^; AcandisGmbH & Co. KG; Pforzheim; Germany) by direct common carotid artery puncture due to severe tortuosity of supra-aortic trunks.

## 1. Introduction

Endovascular treatment of intracranial aneurysms is becoming widely available and nowadays has become the first choice of treatment in many centers, especially for older patients with high surgical risks [1,2,3]. This approach usually has shorter procedural times and is commonly associated with shorter recovery and hospitalization times compared to the traditional neurosurgical approach [4]. This shift towards endovascular procedures has been increasing since the introduction of new generation flow diverter stents (FDS) to the market, which allow for the treatment of wide necked aneurysms [5]. FDS involve the indirect reconstruction of the affected arterial segment rather than direct obliteration of the aneurysm.

Although risks of endovascular procedures for unruptured aneurysms are generally low [6], the presence of severe vessel tortuosity can lead to difficulty in catheterization and therefore complications on the vessels or failure of treatment. The possibility of using different vascular access rather than the classic femoral artery, such as the radial or carotid ones, could help reduce this failure rate.

We present the first reported Italian case of a DERIVO^®^ Flow Diverter stent (FDS) placement in a wide necked intracranial aneurysm by direct carotid puncture.

## 2. Case Presentation

**Figure 1:**
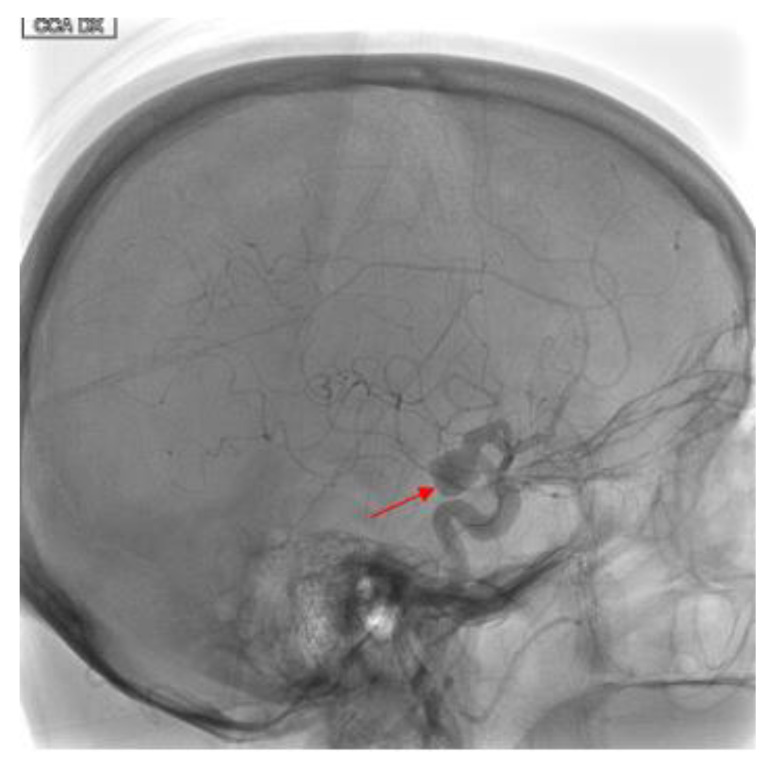
Eighty-year-old female with right internal carotid artery sacciform aneurysm. Findings: Latero-lateral view of digital subtracted angiography demonstrates a medium-sized, wide-necked, sacciform aneurysm artery (solid arrow) at the sovra-clinoid tract of the right internal carotid. Technique: DSA low-dose, 80 Kv, 14 mAs; 2 frame × second (4 s) 1 frame × second (8 s).

In March 2018 an 80-year-old woman with mild cognitive impairment and migraine was admitted to our University Hospital due to the incidental finding of a saccular aneurysm of the right internal carotid artery at the sovra-clinoid segment. She had history of breast cancer as well as arterial hypertension and dyslipidemia. The aneurysmatic lesion was discovered with a brain MRI study, which she underwent due to worsening of her cognitive performances. At a clinical examination, there were no signs of neurological deficits or symptoms compatible with the presence of the aneurysm. The patient underwent a digital subtracted angiography (DSA) to better evaluate the size and morphology of the lesion. DSA showed the presence of a medium-sized, wide-necked, sacciform aneurysm (measuring 10 × 11 mm) of the right internal carotid artery (ICA) at the sovra-clinoid tract (C5), associated with a small wall ectasia of the communicating tract (C7) (measuring 2 mm) (Figure 1). A severe tortuosity of supra-aortic vessels was witnessed, with no other aneurysmatic lesions or vascular malformations of the intracranial circulation. The choice for an endovascular approach was madeduring a multidisciplinary meeting held by neurosurgeons and neuroradiologists, taking into account the size, the anatomical location of the aneurysm as well as patients age and comorbidities. The patient accepted to undergo the endovascular treatment by deployment of a FDS (“DERIVO^®^” Embolization Device, Acandis, Pforzheim, Germany) which was planned for the next month. Informed patient consent for the procedure and for publication was obtained. 

The patient was premedicated with double antiplatelet therapy with clopidogrel 75 mg/day and acetylsalicylic acid 100 mg/day one-week prior the operation. 

**Figure 2:**
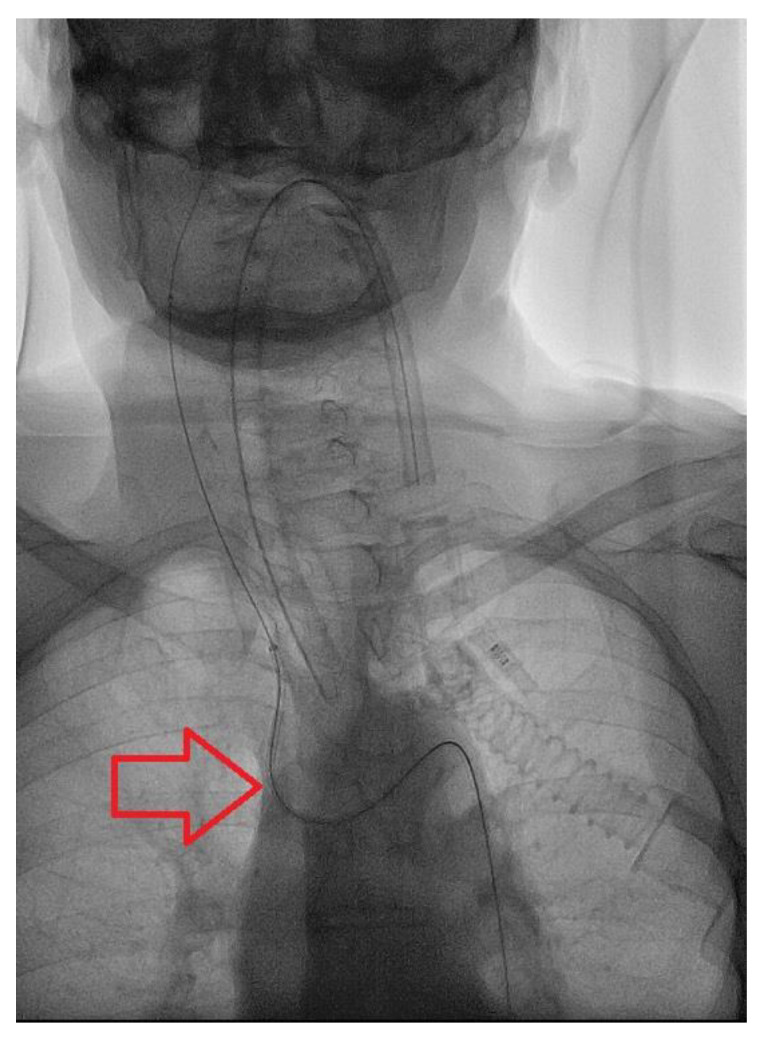
Findings: Antero-posterior view during catheterization of the anonymous trunk shows type-3 Aortic Arch. Technique: Pulsed Fluoroscopy; 15 pulses per second.

**Figure 3:**
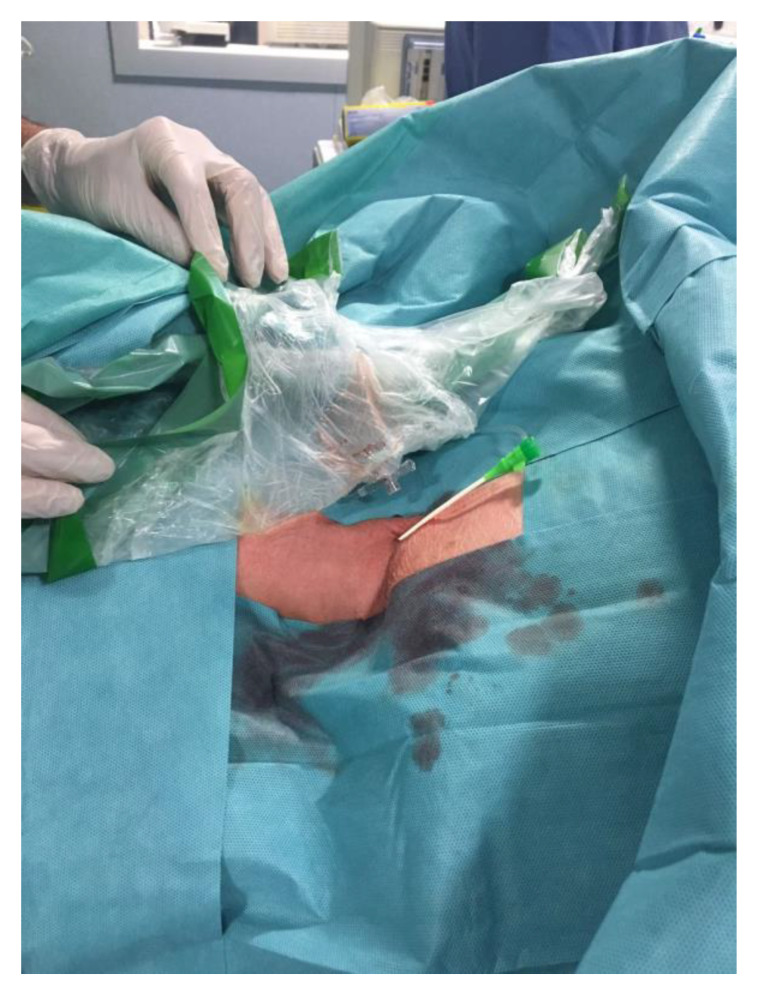
6-French vascular sheath placed in the right common carotid artery after direct vessel puncture with ultrasound guide.

**Figure 4:**
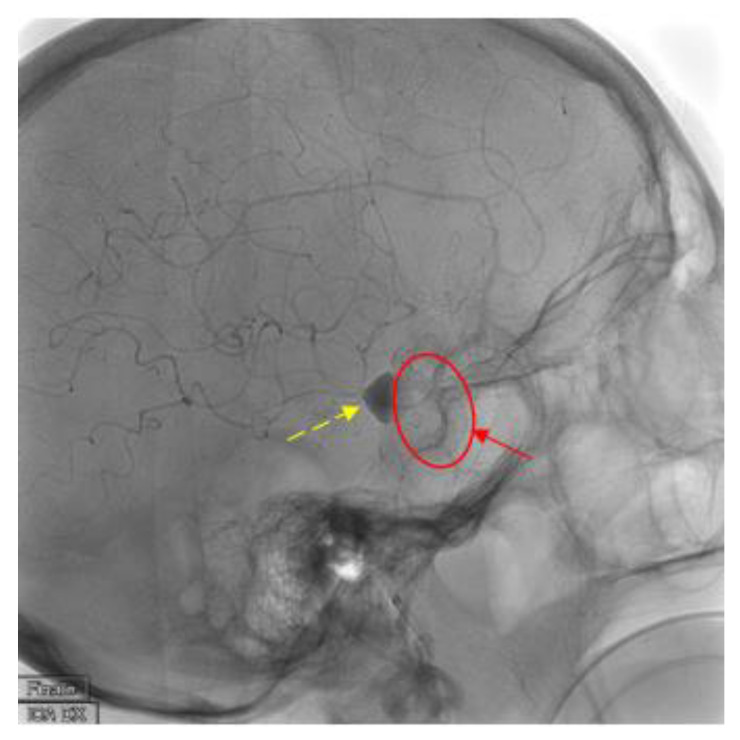
Findings: Latero-lateral view of digital subtracted angiography demonstrates correct placement of the flow-diverter stent (solid arrow and circle) with complete coverage of the aneurysm vessel and contrast medium stasis inside the aneurysm sac (dashed line arrow). Technique: DSA low-dose, 80 Kv, 14 mAs; 2 frame × second (4 s) 1 frame × second (8 s).

During the preoperative phase of DSA, due to the tortuosity of the vessels (Type 3 aortic arch) (Figure 2), it was not possible to place a stable guiding catheter in the right ICA, leading to a severe instability of the triaxial system while navigating the artery. Therefore, we proposed a different approach to the lesion, by direct puncture of the right common carotid artery(CCA) with ultrasound guide, (Figure 3) using a short 6-French sheath and a coaxial system with Neuron 053’’ (Penumbra Inc., Alameda, CA, USA) as guiding catheter and Excelsior XT-27 (StrykerNeurovascular, Fremont, CA, USA) as microcatheter to deliver the stent. We managed to successfully deploy the FDS “DERIVO 5,5 × 20 mm” covering the aneurysmatic lesion (Figure 4). During the procedure, we administered the patient 5000 UI of heparin, after carotid puncture and placement of the vascular sheath, and 500 mg of acetylsalicylic acid after stent deployment. An additional 2000 UI of heparin was administered at the end of the procedure. The patient was then sent to the operating theater were the vascular surgeon performed surgical repair of the CCA after the vascular sheath removal.

After surgical repair of the CCA, the patient was woken up showing no neurological deficits. During post-operative monitoring, about six hours after the endovascular procedure, however, she complained of swelling of the neck and therefore a second surgical review of the carotid access was required. No other post-procedural complications were witnessed, and the patient was discharged four days after the procedure.

**Figure 5:**
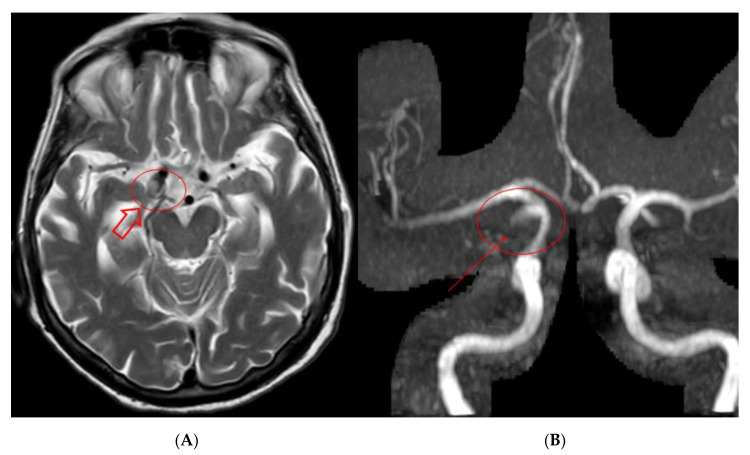
Findings: Six-months MR and MRA performed at our institution: (**A**) T2 brain acquisition showing shrinkage of the aneurysmatic sac on the left ICA (hollow arrow); (**B**) 3D TOF MRA acquisition showing minimal signal intensity on the neck portion of the aneurysmatic sac (solid arrow).

Follow-up was performed at 6 (Figure 5), 12 and 24 months with Angio-MR with 3d-TOF and CEMRA technique showing complete exclusion of the aneurysmatic lesion at 24 months without any delayed complications. At follow-up the patient remains asymptomatic.

All procedures performed in studies involving human participants were in accordance with the 1964 Helsinki Declaration and its later amendments or comparable ethical standards.

## 3. Discussion

Endovascular treatment of intracranial aneurysm is becoming the first treatment of choice especially in older patient with high surgical risk. This approach has usually shorter procedural times and is commonly associated with shorter recovery and hospitalization times compared to the traditional neurosurgical approach.

One of the main challenges of endovascular treatment is represented by the catheterization of the affected artery, which, especially in older patients, can be difficult due to tortuosity of the vessels. Many authors described different approaches for neurointerventional procedures, such as brachial and radial access [7]; these approaches have a lower risk rate during hemostatic closure, but catheterization of the common carotid artery is not always simple through this approach, since it usually arises with a steep angle from the aortic arch, which may cause drop or kinking of the guiding catheter. 

Direct carotid puncture, although presenting a higher risk of bleeding and neck hematoma, allows us to skip the unfavorable aortic arch anatomy with faster procedural times and lower risks of complication on the vessels.

In our case the bleeding risk was increased as the patient was under double antiplatelet therapy. It was necessary to puncture the neck under ultrasound guidance at the level of the common carotid artery about one centimeter lower than the bifurcation, to leave enough space for the surgeon during the surgical repair of the vessel. The antiplatelet therapy was not considered a contraindication to vascular surgery.

Most authors descriptionsof carotid access for neurointerventional procedures are from reports of emergency cases ratherthan elective treatments, such as stroke and ruptured aneurysm coiling [8,9]. In the event of excessive tortuosity of the supra-aortic trunks or inability to use the iliac axes due to severe atheromasia, the possibility of direct access to the carotid artery may represent an important option in this type of patient.

Direct carotid puncture under ultrasonography guidance have been reported in a few case series, that reported good outcomes with fast procedural times [9,10,11]. Lee et al. reported a series of 17 patients that were treated with neurointerventional procedures by direct carotid approach. All patients in the series were treated by exposure of the carotid artery in hybrid endovascular theater with surgical exposure of the vessel, no complications were reported, but procedural times were not compared to the percutaneous puncture [12]. 

Another study from Larrazabal et al. reports a series of 4 patients that underwent neuroendovascular procedures (cerebral aneurysm coiling in 3 patients and retrograde common carotid artery stenting in one) by surgical exposure of the carotid artery and subsequent direct arterial puncture; in this study one patient was treated with only local anesthesia and no complications related to the vascular access were reported [13]. 

Dorfer et al. reported a comparison of 23 patients treated with both direct percutaneous puncture and surgical exposure, which showed similar results, without complication in both the percutaneous and surgical access [14].

No closing devices are currently approved for percutaneous carotid puncture; Cuellar et al. reported a case series of eight patients were Angioseal™ (Vascular Closure Device, Terumo, Tokyo, Japan) was used to perform carotid hemostasis, reporting no device related complications; the main weakness of this study is the small sample size, therefore all closing devices used with this vascular access are still off-label [15].

Only one other case of flow diverter positioned by direct puncture of the carotid artery is reported in the literature; in that case a giant cavernous carotid internal aneurysm was treated in 2019; in this case report the patient had bilateral mirror aneurysms of the cavernous segment but was treated by direct carotid puncture only on the left side; they also performed percutaneous ultrasound guided puncture of common carotid artery but hemostasis was obtained by deployment of MynxGrip collagen plug device (AccessClosure, Inc., Mountain View, CA, USA) with no registered complications [16].

Choice of vascular access closure was taken together with the vascular surgeon, taking into account the fact that we did not have Angioseal nor MynxGrip available at the time of the procedure, and the only closure device available (PerClose ProGlide ™, Abbott Laboratories, Chicago, IL, USA) was considered to be too large and was not reported in literature for carotid artery closure.

In our experience, the surgical closure of the percutaneous carotid puncture appeared to be slightly more complicated than the surgical repair of the carotid artery punctured after exposure, but it is a procedure that can be performed also in a regular angio-suite, with a second time in surgical theater, reducing time of the endovascular procedure. By this approach, choice of carotid access can be delayed after a first attempt of vascular catheterization from the classic femoral access, reserving the carotid puncture as a last resort of vascular access.

## 4. Conclusions

In our experience, percutaneous direct carotid puncture is a viable alternative access and appears to be a safe and effective approach to treat patients with severe supra-aortic vessels tortuosity, which prevents the traditional femoral approach. Use of ultrasound, in our opinion, is a great tool to reduce puncture risks and to correctly position the vascular sheath. Hemostasis can be complicated to obtain; since no closure device has been approved for carotid puncture each operator should choose according to one’s experience, not forgetting that surgical closure is still a viable option. We reported as vascular access complication a minor bleeding of the surgical wound, which required surgical revision but did not affect patient neurological conditions nor did it prolong hospitalization times.

## References

[1] Molyneux A.J., Kerr R.S.C., Stratton I., Sandercock P., Clarke M., Shrimpton J., Holman R., International Subarachnoid Aneurysm Trial (ISAT) Collaborative Group (2002). International Subarachnoid Aneurysm Trial (ISAT) of neurosurgical clipping versus endovascular coiling in 2143 patients with ruptured intracranial aneurysms: A randomised trial. Lancet.

[2] Molyneux A.J., Kerr R.S.C., Birks J., Ramzi N., Yarnold J., Sneade M., Rischmiller J., ISAT Collaborators (2009). Risk of recurrent subarachnoid haemorrhage, death, or dependence and standardised mortality ratios after clipping or coiling of an intracranial aneurysm in the International Subarachnoid Aneurysm Trial (ISAT): Long-term follow-up. Lancet Neurol..

[3] McDougall C.G., Spetzler R.F., Zabramski J.M., Partovi S., Hills N.K., Nakaji P., Albuquerque F.C. (2012). The Barrow Ruptured Aneurysm Trial. J. Neurosurg..

[4] Brinjikji W., Cloft H.J., Fiorella D., Lanzino G., Kallmes D.F. (2013). Estimating the proportion of intracranial aneurysms likely to be amenable to treatment with the pipeline embolization device. J. Neurointerv. Surg..

[5] Mazur M.D., Taussky P., Park M.S., Couldwell W.T. (2018). Contemporary endovascular and openaneurysm treatment in the era of flow diversion. J. Neurol. Neurosurg. Psychiatry.

[6] Briganti F., Leone G., Marseglia M., Mariniello G., Caranci F., Brunetti A., Maiuri F. (2015). Endovascular treatment of cerebral aneurysms using flow-diverter devices: A systematic review. Neuroradiol. J..

[7] Levy E.I., Boulos A.S., Fessler R.D., Bendok B.R., Ringer A.J., Kim S.H., Qureshi A.I., Guterman L.R., Hopkins L.N. (2002). Transradial cerebral angiography: An alternative route. Neurosurgery.

[8] Wiesmann M., Kalder J., Reich A., Brockmann M.A., Othman A., Greiner A., Nikoubashman O. (2016). Feasibility of combined surgical and endovascular carotid access for interventional treatment of ischemic stroke. J. Neurointerv. Surg..

[9] Mokin M., Snyder K.V., Levy E.I., Hopkins L.N., Siddiqui A.H. (2015). Direct carotid artery puncture access for endovascular treatment of acute ischemic stroke: Technical aspects, advantages, and limitations. J. Neurointerv. Surg..

[10] Nii K., Kazekawa K., Onizuka M., Aikawa H., Tsutsumi M., Tomokiyo M., Iko M., Kodama T., Matsubara S., Go Y. (2006). Direct carotid puncture for the endovascular treatment of anterior circulation aneurysms. Am. J. Neuroradiol..

[11] Matsuda Y., Terada T., Masuo O., Matsumoto H., Ohshima K., Tsumoto T., Tsuura M. (2013). The clinical results of transcervical carotid artery stenting and frequency chosen as the approach route of carotid artery stenting in 1,067 consecutive cases. Acta Neurochir..

[12] Lee J.Y., Park J.H., Jeon H.J., Yoon D.Y., Park S.W., Cho B.M. (2018). Transcervical access via direct neck exposure for neurointerventional procedures in the hybrid angiosuite. Neuroradiology.

[13] Larrazabal R., Klurfan P., Sarma D., Gunnarsson T. (2010). Surgical exposure of the carotid artery for endovascular interventional procedures. Acta Neurochir..

[14] Dorfer C., Standhardt H., Gruber A., Ferraz-Leite H., Knosp E., Bavinzski G. (2012). Direct Percutaneous Puncture Approach versus Surgical Cutdown Technique for Intracranial Neuroendovascular Procedures: Technical Aspects. World Neurosurg..

[15] Cuellar H., Guimaraens L., Ambekar S., Vivas E., Theron J. (2015). Angioseal as a hemostatic device for direct carotid puncture during endovascular procedures. Interv. Neuroradiol..

[16] Brunozzi D., Shakur S.F., Alaraj A. (2019). Pipeline Embolization of Giant Cavernous Internal Carotid Artery Aneurysm with Direct Carotid Puncture and Arteriotomy Closure Device: Neuroendovascular Surgical Video. World Neurosurg..

